# Supplementary material for: Nonuniform and pathway-specific laminar processing of spatial frequencies in the primary visual cortex of primates
Source: Nat Commun. 2024 May 13;15:4005. doi: 10.1038/s41467-024-48379-x (PMC11091180; doi:10.1038/s41467-024-48379-x)
Supplement: Supplementary file 1 — Supplementary Information [file 41467_2024_48379_MOESM1_ESM.pdf]

Nonuniform and pathway-specific laminar processing of spatial frequencies in the primary visual cortex of primates

Supplementary Information

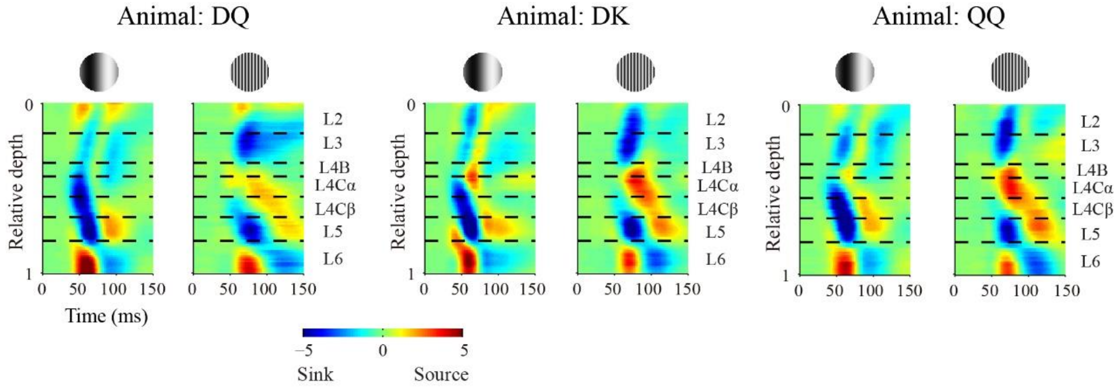

Supplementary Fig. 1 Laminar assignments of the cortical layers of individual animals. The laminar patterns of the current source density (CSD) for low (1.25 cycles/degree) and high SFs (10 cycles/degree) were averaged over all probe placements in individual animals (DQ, DK and QQ). CSD was coded by color. The horizontal dashed lines indicate the laminar boundaries in V1. Source data are provided as a Source Data file.

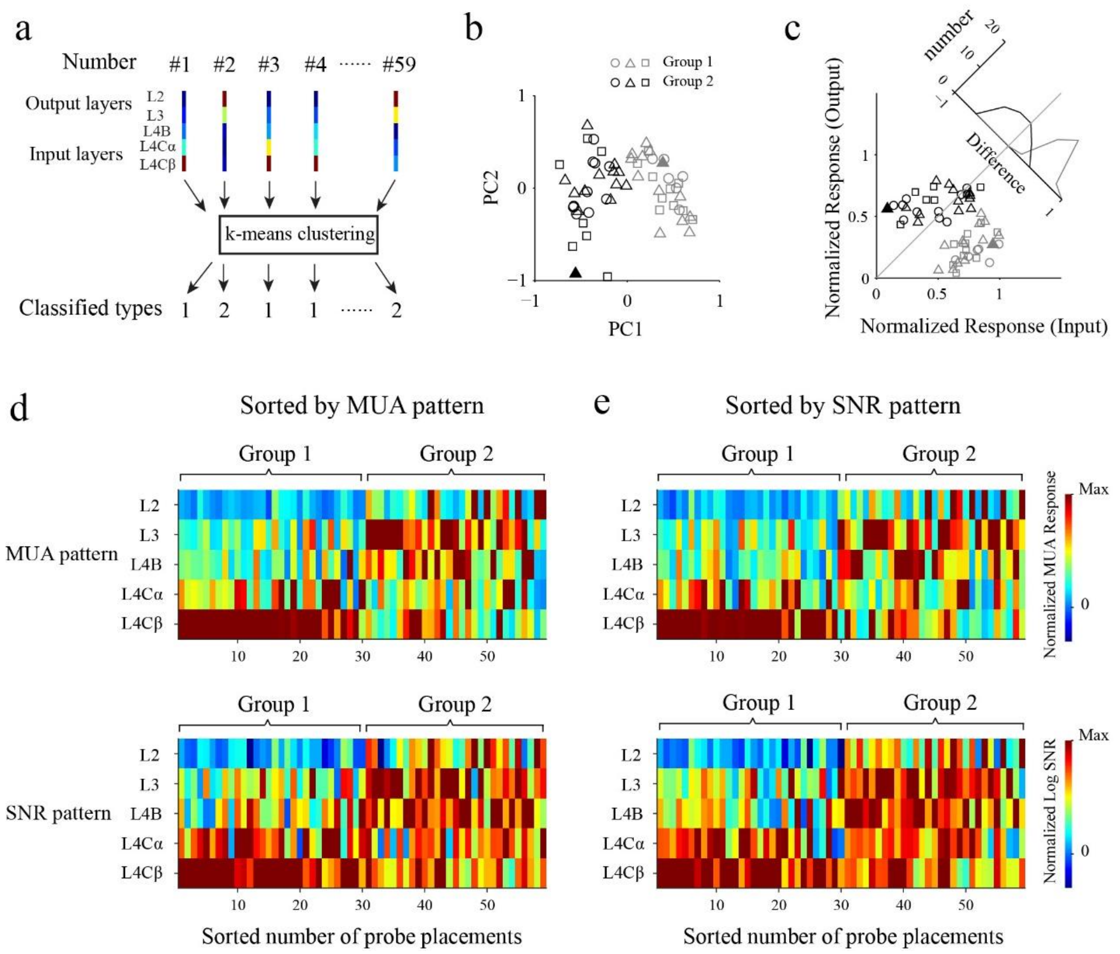

Supplementary Fig. 2 Grouping analysis results obtained based on the k-means clustering algorithm. a Illustration of k-means clustering (see Methods for details). **b** Scatter plot (n = 59 probe placements) of the joint distribution of the first two components of a principal component analysis (PCA). The gray dots represent group 1, and the black dots represent group 2. The different shapes represent the three animals (DQ: circles; DK: triangles; QQ: squares). **c** Scatter plot (n = 59 probe placements) of the normalized responses of the input layers and output layers. Gray represents group 1, and black represents group 2. The histogram shows the difference between the responses of the input and output layers. The filled triangles in **b** and **c** were calculated from the data obtained with probe placements shown in Figure 1e. **d** Patterns derived from all probe placements and grouped by their MUA patterns. The probe placements were sorted by their distances from the center of the first group. The upper panel represents the MUA patterns. The lower panel represents the signal-to-noise ratio (SNR) patterns. Log indicates the base-10 logarithm. The MUA and SNR values were normalized by the maximum values determined across all layers. **e** Similar to **d** but for results grouped by SNR patterns. The results obtained by grouping according to the SNR patterns were similar to those of the MUA responses, which suggests that our clustering results were robust. Source data are provided as a Source Data file.

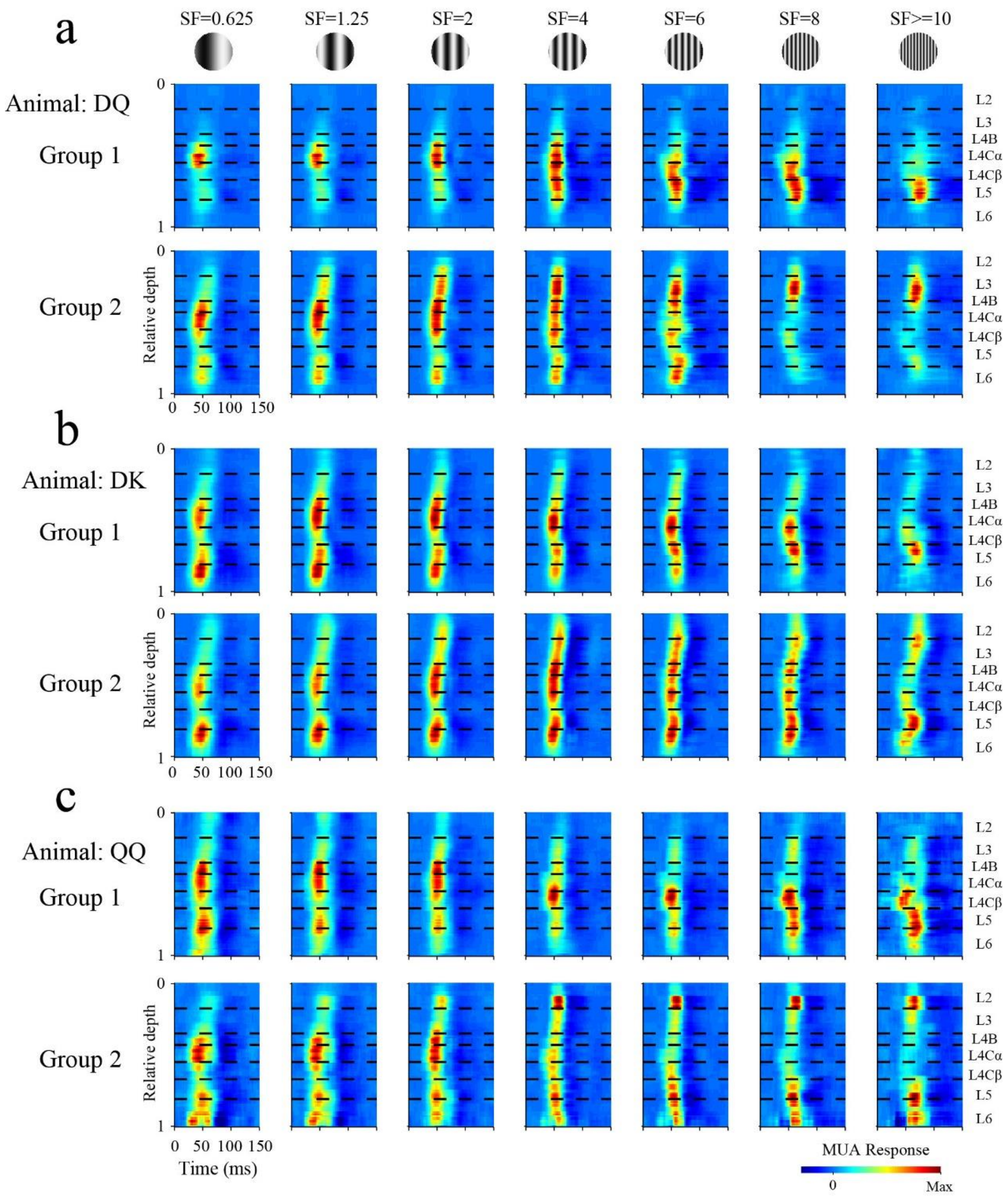

Supplementary Fig. 3 Laminar patterns of individual animals. a Population-averaged laminar pattern for one animal (DQ). The two groups are presented separately (upper panels for group 1 and lower panels for group 2). The strengths of the MUA responses are indicated by color. The length of the sliding window for averaging across the depth dimension was 0.1 (relative depth). The horizontal black dashed lines represent the laminar boundaries. Each SF response pattern was normalized by dividing it by its maximum value. **b** and **c** Similar to **a** but for the other two animals (DK and QQ, respectively). Source data are provided as a Source Data file.

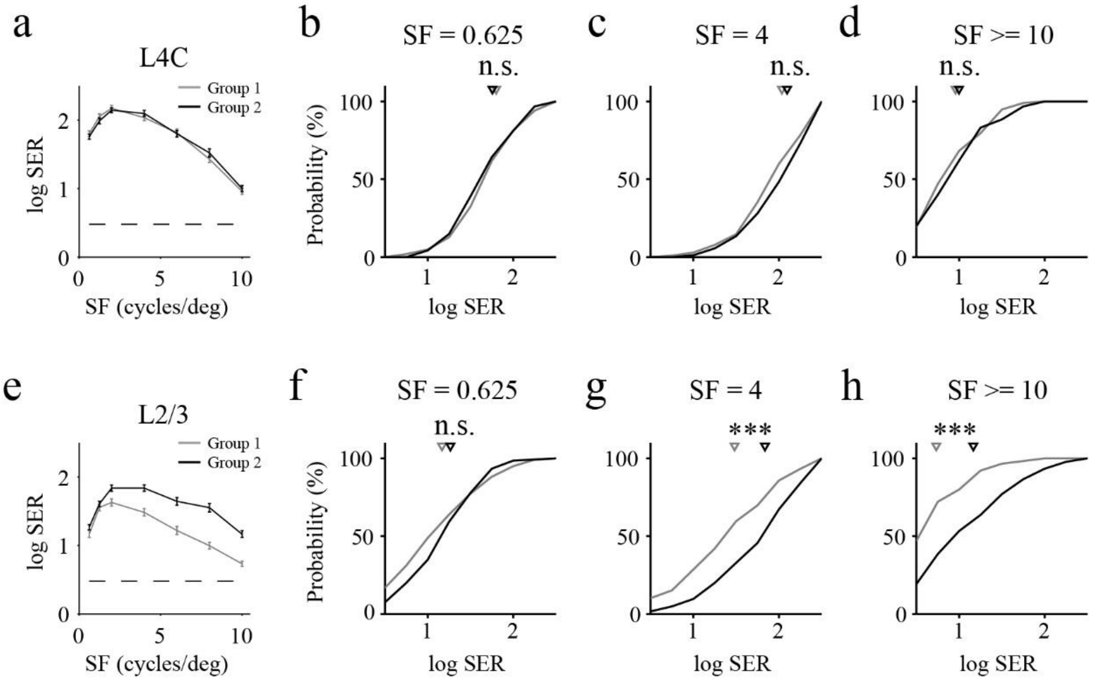

Supplementary Fig. 4 Both groups could be effectively activated by visual stimuli. a Log SER values as functions of the SFs in L4C for the two groups. The stimulus-driven energy ratio (SER) was used to select visually driven sites (see Methods for details). Gray represents group 1, and black represents group 2. Log indicates the base-10 logarithm. The horizontal black dashed lines represent the threshold (SER = 3). Data are presented as mean values ±SEM, n = 30 probe placements for Group 1, n = 29 probe placements for Group 2. **b-d** Cumulative probability distributions of the log SER for L4C. **b** SF = 0.625. **c** SF = 4. **d** SF ≥ 10. The line colors represent two groups. Two-sided rank-sum test (n = 30 probe placements for Group 1, n = 29 probe placements for Group 2; for SF = 0.625, p = 0.43; for SF = 4, p = 0.3; for SF ≥ 10, p = 0.61). **e-h** Similar to **a-d** but for L2/3. Two-sided rank-sum test (n = 30 probe placements for Group 1, n = 29 probe placements for Group 2; for SF = 0.625, p = 0.1; for SF = 4, $p<10^{-5}$; for SF ≥ 10, $p<10^{-9}$). Source data are provided as a Source Data file.

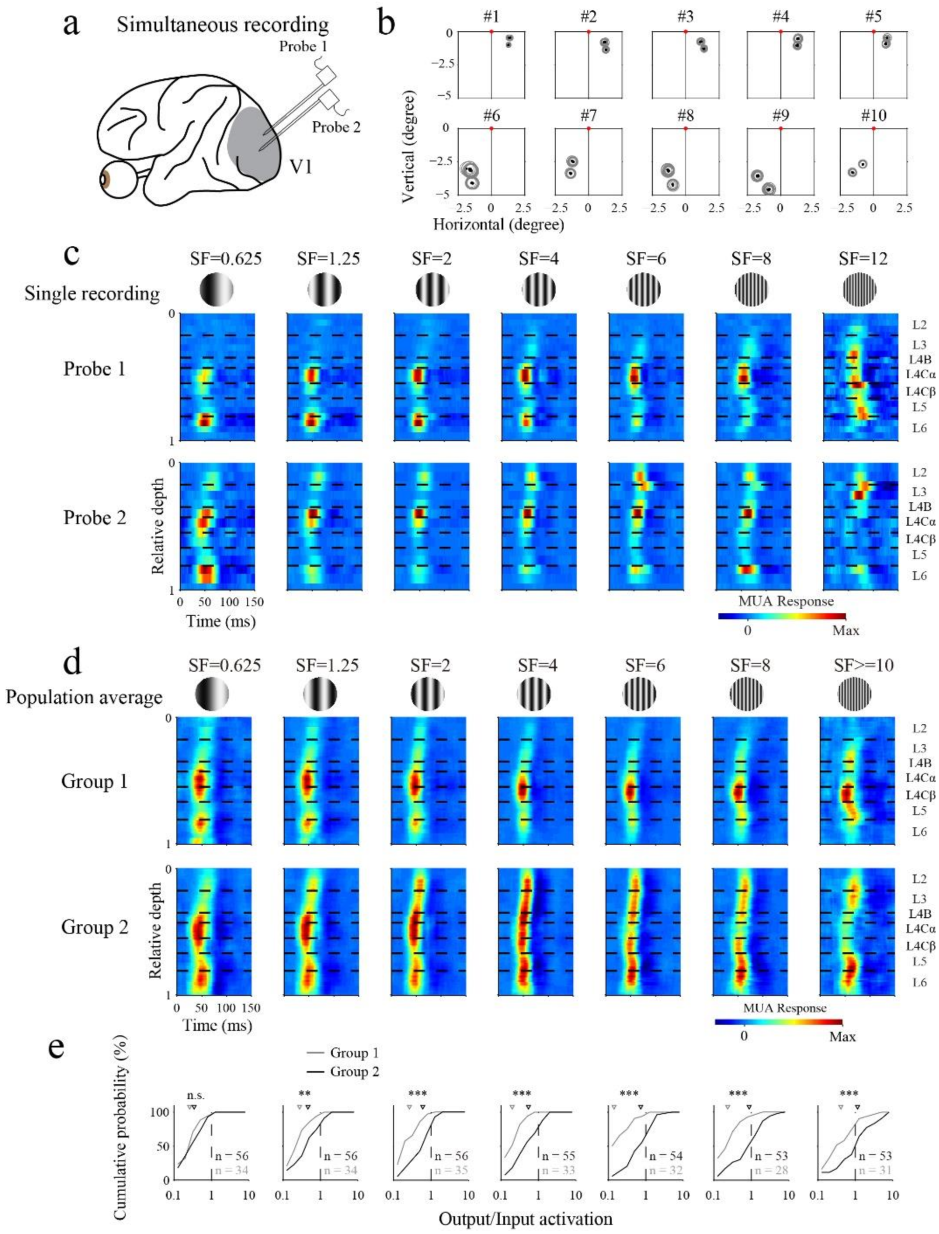

Supplementary Fig. 5 Two groups of laminar processing patterns induced by SFs were also observed for simultaneously recorded probe placements. **a** Two V-probes (Plexon, 24 channels, interchannel spacing of 100 μm) were simultaneously inserted into V1 (gray region) to record neural responses from all layers. **b** Spatial distributions for the RF centers (dots) of sites recorded from two simultaneously inserted probes. Each subfigure represents a single recording (ten simultaneous recordings in total). The circles represent the locations (centers) and sizes (radii) of the RFs. The black dots represent the RF center of each site. **c** Laminar response patterns of the MUAs derived from two probe placements in a single recording (the upper panels show probe 1, and the lower panels show probe 2). The horizontal black dashed lines represent the laminar boundaries. Each SF response pattern was normalized by dividing it by its maximum value. **d** Population-averaged laminar patterns for ten simultaneous recordings (20 probe placements). The upper panels show group 1 (n = 8), and the lower panels show group 2 (n = 12). The length of the sliding window for averaging across the depth dimension was 0.1 (relative depth). **e** Cumulative probability distributions of the output/input activation for L2/3. For each SF condition, sites with responses higher than 0 were included (n is the number of valid sites). Two-sided rank-sum test, p value for seven SF conditions: $p=0.21$, $p=0.004$, $p<10^{-5}$, $p<10^{-5}$, $p<10^{-7}$, $p<10^{-5}$, $p<10^{-3}$. Different line colors represent the two groups. ***$p < 0.001$, **$p < 0.01$, n.s., not significant. Source data are provided as a Source Data file.

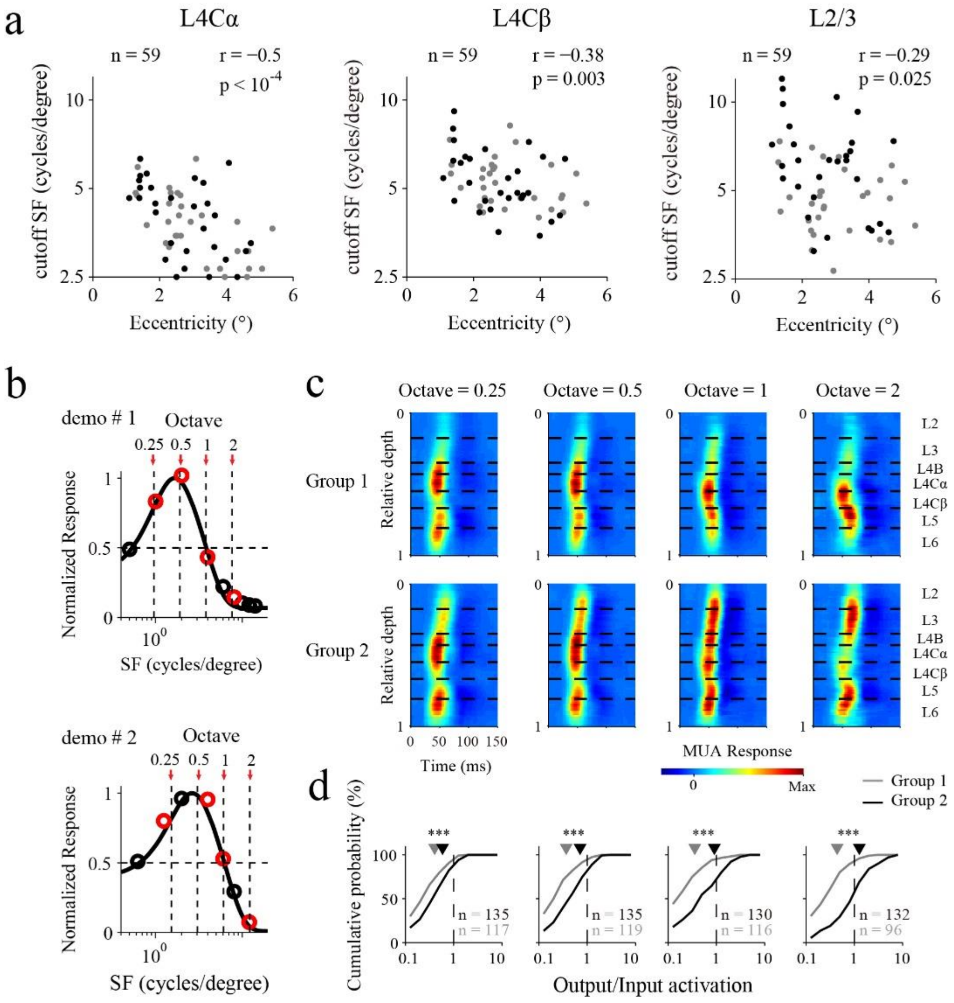

Supplementary Fig. 6 Laminar response patterns averaged from corrected SFs were similar to those shown in Fig. 2. a Relationship between the cutoff SF and eccentricity (n = 59 probe placements). Each dot represents data averaged from all sites at the same probe placement within each layer. Gray represents group 1, and black represents group 2. The first column denotes L4Cα, the second column represents L4Cβ, and the third column signifies L2/3 (r is the Pearson's correlation coefficient). **b** Example recording sites of L4Cβ for two probe placements. The red arrows and vertical dashed lines represent SFs relative to the cutoff SF (4 levels: 0.25, 0.5, 1, 2 octaves). The red dots represent the SFs of the stimuli nearest to these 4 levels. The horizontal dashed lines represent 0.5. **c** Population-averaged laminar patterns for different SF levels. The two groups are presented separately (the upper panels show group 1, and the lower panels show group 2). The strength of MUA response is indicated by its color. The length of the sliding window for averaging across the depth dimension was 0.1 (relative depth). The horizontal black dashed lines represent the laminar boundaries. Each pattern of the SF response was normalized by dividing it by its maximum value. **d** Cumulative probability distributions of the output/input activations for L2/3. For each probe placement, the responses of the input layers were averaged across all sites of L4Cα and L4Cβ. The responses were averaged from 0 to 120 ms after the onset of the stimulus. For each SF condition, sites with responses higher than 0 were included (n is the number of valid sites). Two-sided rank-sum test, $p < 10^{-3}$ for 0.25 octaves, $p < 10^{-7}$ for 0.5 octaves, $p < 10^{-9}$ for 1 octaves, $p < 10^{-14}$ for 2 octaves. Gray represents group 1, and black represents group 2. ***$p < 0.001$. The average values are indicated by triangles. Source data are provided as a Source Data file.

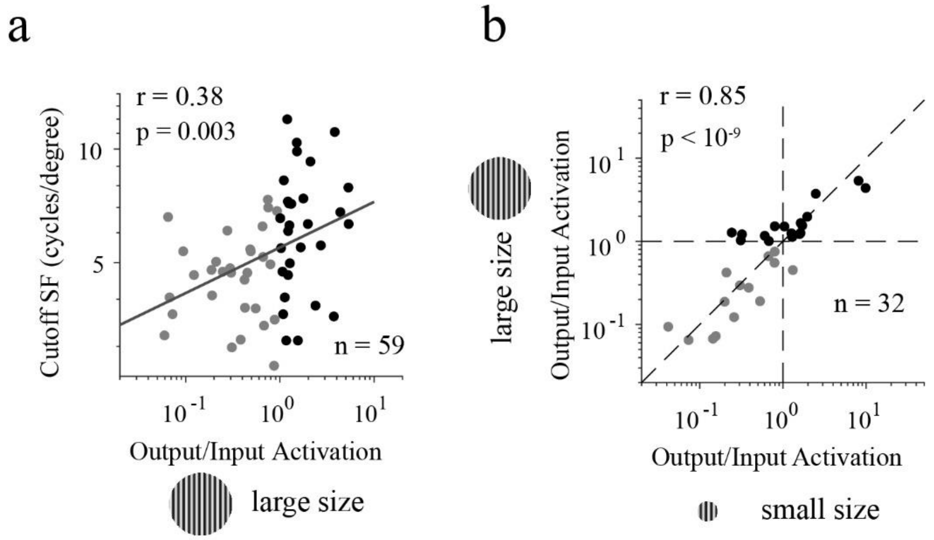

Supplementary Fig. 7 Relationships between the output/input activation and the other parameters. a Relationship between the output/input activation and the cutoff SFs. The gray dots represent group 1, the black dots represent group 2, and r is the Pearson's correlation coefficient (n = 59 probe placements, r = 0.38, p = 0.003). **b** Relationship between the output/input activations calculated from small and large stimuli under the high SF condition. The gray dots represent group 1, the black dots represent group 2, and r is the Pearson's correlation coefficient (n = 32 probe placements, r = 0.85, $p < 10^{-9}$). Source data are provided as a Source Data file.

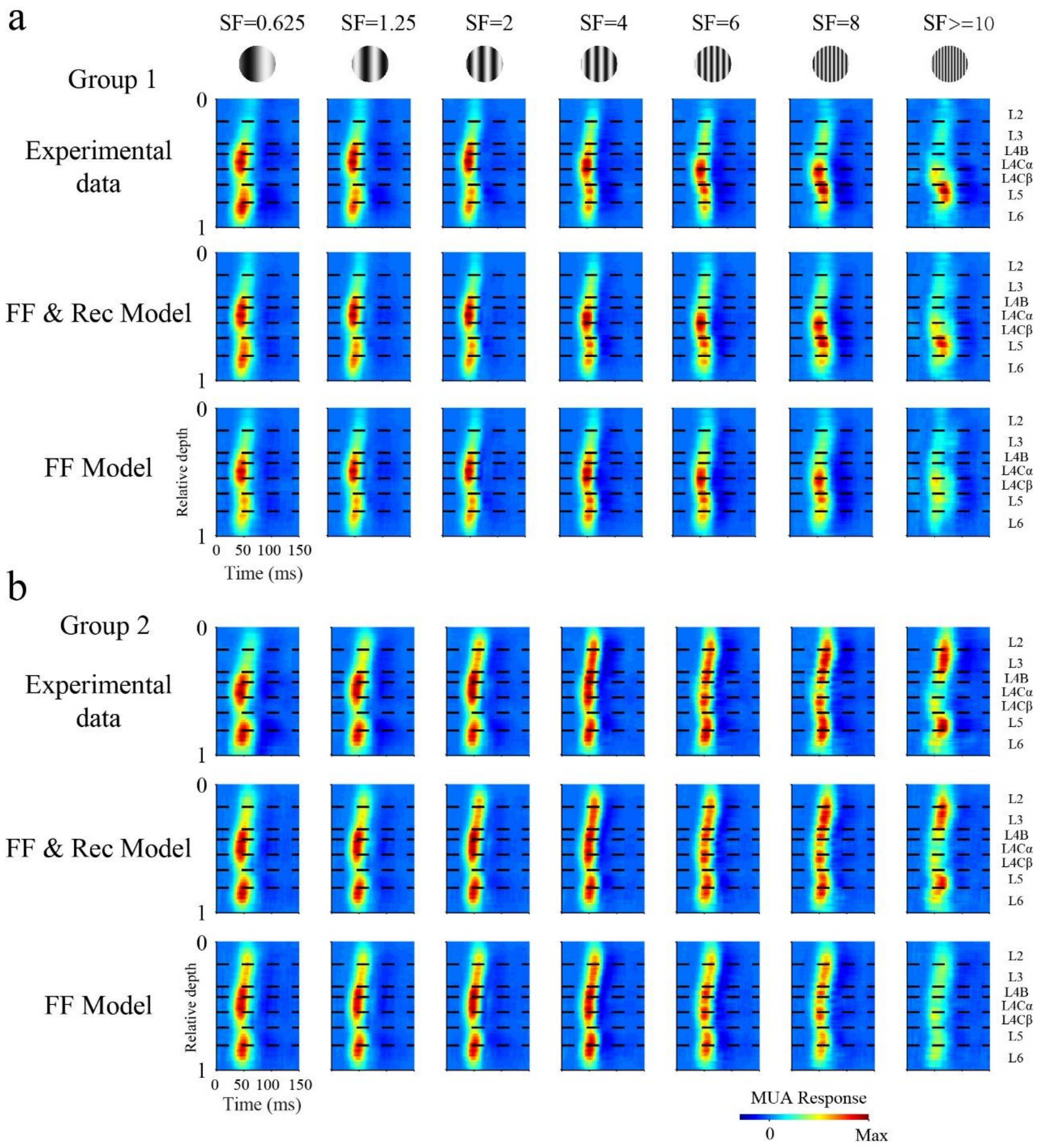

Supplementary Fig. 8 Laminar patterns can be explained by two feedforward components derived from the M and P pathways and one recurrent component. a Population-averaged model fitting results obtained for group 1. Top panel: experimental data; middle panel: model fitting results produced for the FF & Rec model; bottom panel: model fitting results produced for the FF model. The response pattern for each SF was normalized by dividing it by the corresponding peak value of the response in the experimental data. The horizontal black dashed lines represent the laminar boundaries. The strength of MUA response is indicated by its color. **b** Similar to **a** but for the results of group 2. Source data are provided as a Source Data file.

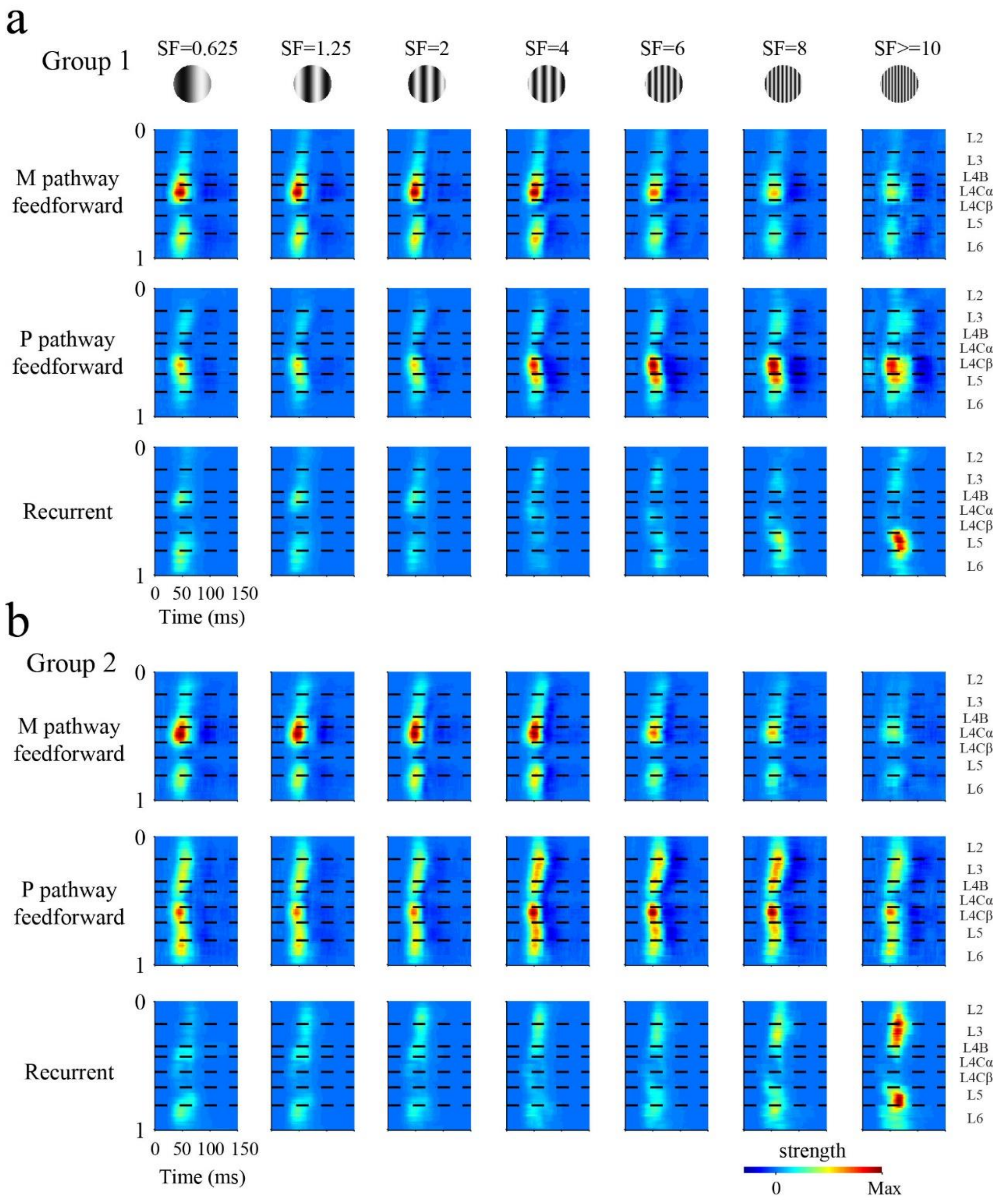

Supplementary Fig. 9 Population-averaged laminar patterns of the three components. a Population-averaged laminar patterns of the three components for group 1. Top panel: feedforward component of the M pathway; middle panel: feedforward component of the P pathway; bottom panel: recurrent component. The response pattern for each SF was normalized by dividing it by the maximum value among the three components. The horizontal black dashed lines represent the laminar boundaries. The strength of components is indicated by its color. **b** Similar to **a** but for the results of group 2. Source data are provided as a Source Data file.
