# Supplementary material for: Nonuniform and pathway-specific laminar processing of spatial frequencies in the primary visual cortex of primates
Source: Nat Commun. 2024 May 13;15:4005. doi: 10.1038/s41467-024-48379-x (PMC11091180; doi:10.1038/s41467-024-48379-x)
Supplement: Supplementary file 3 — Reporting Summary [file 41467_2024_48379_MOESM3_ESM.pdf]

nature portfolio

Corresponding author(s): Dajun Xing

Last updated by author(s): Apr 1, 2024

Reporting Summary

Nature Portfolio wishes to improve the reproducibility of the work that we publish. This form provides structure for consistency and transparency in reporting. For further information on Nature Portfolio policies, see our Editorial Policies and the Editorial Policy Checklist.

Statistics

For all statistical analyses, confirm that the following items are present in the figure legend, table legend, main text, or Methods section.

n/a	Confirmed	
☐	☒	The exact sample size (*n*) for each experimental group/condition, given as a discrete number and unit of measurement
☐	☒	A statement on whether measurements were taken from distinct samples or whether the same sample was measured repeatedly
☐	☒	The statistical test(s) used AND whether they are one- or two-sided<br>*Only common tests should be described solely by name; describe more complex techniques in the Methods section.*
☐	☒	A description of all covariates tested
☒	☐	A description of any assumptions or corrections, such as tests of normality and adjustment for multiple comparisons
☐	☒	A full description of the statistical parameters including central tendency (e.g. means) or other basic estimates (e.g. regression coefficient) AND variation (e.g. standard deviation) or associated estimates of uncertainty (e.g. confidence intervals)
☐	☒	For null hypothesis testing, the test statistic (e.g. *F*, *t*, *r*) with confidence intervals, effect sizes, degrees of freedom and *P* value noted<br>*Give P values as exact values whenever suitable.*
☒	☐	For Bayesian analysis, information on the choice of priors and Markov chain Monte Carlo settings
☒	☐	For hierarchical and complex designs, identification of the appropriate level for tests and full reporting of outcomes
☐	☒	Estimates of effect sizes (e.g. Cohen's *d*, Pearson's *r*), indicating how they were calculated

Our web collection on statistics for biologists contains articles on many of the points above.

Software and code

Policy information about availability of computer code

Data collection	Raw data were acquired by a 128-channel system (Blackrock Microsystems).
Data analysis	For all data analyses we used standard MATLAB functions (e.g. convn, fmincon etc; version:R2013a). The custom MATLAB functions and scripts used to produce the results presented in this study are publicly available via GitHub: https://github.com/BNUTW2023/PSLPSFV1 and deposited at https://zenodo.org/records/10890515.

For manuscripts utilizing custom algorithms or software that are central to the research but not yet described in published literature, software must be made available to editors and reviewers. We strongly encourage code deposition in a community repository (e.g. GitHub). See the Nature Portfolio guidelines for submitting code & software for further information.

Data

Policy information about availability of data

All manuscripts must include a data availability statement. This statement should provide the following information, where applicable:

- Accession codes, unique identifiers, or web links for publicly available datasets
- A description of any restrictions on data availability
- For clinical datasets or third party data, please ensure that the statement adheres to our policy

The data used in the figures in this study are provided in a source data file. The dataset underlying the results can be found in https://github.com/BNUTW2023/PSLPSFV1 and deposited at https://zenodo.org/records/10890515. Source data are provided with this paper.

Research involving human participants, their data, or biological material

Policy information about studies with human participants or human data. See also policy information about sex, gender (identity/presentation), and sexual orientation and race, ethnicity and racism.

Reporting on sex and gender	N/A
Reporting on race, ethnicity, or other socially relevant groupings	N/A
Population characteristics	N/A
Recruitment	N/A
Ethics oversight	N/A

Note that full information on the approval of the study protocol must also be provided in the manuscript.

Field-specific reporting

Please select the one below that is the best fit for your research. If you are not sure, read the appropriate sections before making your selection.

☒ Life sciences ☐ Behavioural & social sciences ☐ Ecological, evolutionary & environmental sciences

For a reference copy of the document with all sections, see nature.com/documents/nr-reporting-summary-flat.pdf

Life sciences study design

All studies must disclose on these points even when the disclosure is negative.

Sample size	Our sample sizes are chosen based on our previous publications (Wang et al.,2020; Yang et al., 2022). We did electrophysiological recording on three monkeys for all experiments (sparse noise, large gratings with different spatial frequencies, small gratings with different spatial frequencies). There are 59 sessions for the experiment using gratings with large size, 32 sessions for the experiment using gratings with small size. The exact numbers of recording sites have showed in figures or figure legends.
Data exclusions	No data were excluded in the initial analyses.
Replication	We replicated our findings of diverse SF-dependent response patterns in all three monkeys so we think the findings are reproducible.
Randomization	The samples were allocated randomly for all experiment conditions. No experimental groupings were used in this study.
Blinding	Blinding is not relevant because no grouping was performed.

Reporting for specific materials, systems and methods

We require information from authors about some types of materials, experimental systems and methods used in many studies. Here, indicate whether each material, system or method listed is relevant to your study. If you are not sure if a list item applies to your research, read the appropriate section before selecting a response.

Materials & experimental systems

n/a	Involved in the study	
☒	☐	Antibodies
☒	☐	Eukaryotic cell lines
☒	☐	Palaeontology and archaeology
☐	☒	Animals and other organisms
☒	☐	Clinical data
☒	☐	Dual use research of concern
☒	☐	Plants

Methods

n/a	Involved in the study	
☒	☐	ChIP-seq
☒	☐	Flow cytometry
☒	☐	MRI-based neuroimaging

Animals and other research organisms

Policy information about studies involving animals; ARRIVE guidelines recommended for reporting animal research, and Sex and Gender in Research

Laboratory animals	Three male adult rhesus monkeys (Macaca mulatta, 5-7 years, 6-9 Kg).
Wild animals	The study did not involve wild animals.
Reporting on sex	All three monkeys are male. This sample size is too small to make meaningful statements about the effect of sex on the findings that we found. Previous studies of primary visual cortex using macaque monkeys did not reported relevant functional difference between male and female. Since our study focuses on primary visual cortex, differences between sexes are unlikely to our scientific findings.
Field-collected samples	The study did not involve samples collected from field.
Ethics oversight	All procedures were conducted in compliance with the National Institutes of Health Guide for the Care and Use of Laboratory Animals, and were approved by the Institutional Animal Care and Use Committee of Beijing Normal University.

Note that full information on the approval of the study protocol must also be provided in the manuscript.

Plants

Seed stocks	N/A
Novel plant genotypes	N/A
Authentication	N/A
